# Sir Alfred Keogh, G.C.B. M.D.

**Published:** 1918-01-19

**Authors:** 


### SIR ALFRED KEOGH, G.C.B., M.D.,

### Rector of the Imperial College of Science and Technology.

IN THE HOSPITAL of February 10, 1917, page 381, we gave an account of the remarkable career of the Director-General of the Army Medical Services. Sir Alfred Keogh is a great personality. The difficulties which he has had to face all through his War Office career have been heavier than those of any of his predecessors, and his complete success has been wonderful in the circumstances, and entitles him to rank as one of the great administrators of the nation. To-day it is with mingled feelings that we have to announce his retirement from the War Office at the end of February, to enable him to resume his position as Rector of the Imperial College of Science and Technology in London, where his services are urgently called for. Sir Alfred Keogh has our full sympathy in the very difficult circumstances in which he found himself owing to the urgent demands for his whole time, not only in high administrative work at the War Office, but in the field of science at South Kensington. His popularity and splendid service have caused him to return, wholly, to scientific work, with a halo of glory, and the prospect of accomplishing many things of paramount value to the progressive development of the nation and Empire. To-day it will excite widespread interest to give some account of the grounds on which this forecast rests.

### A WISE POLICY OF INITIATIVE.

Among those who have deserved well of the country during the war Surg.-Gen. Sir Alfred Keogh takes a very high place. He made a great reputation during the South African War on account of the knowledge and energy he displayed in coping with outbreaks of typhoid and other diseases among the troops during that campaign, and he secured rapid and well-deserved promotion as Director-General of the R.A.M.C. On his retirement from this position some years later he was appointed the Rector of the Imperial College of Science and Technology, the largest institution of its kind in the Empire. Covering an area of about ten acres of ground at South Kensington, it comprises three constituent colleges, incorporated by Royal Charter in 1907, and is enjoined "to give the highest specialised instruction and to provide the fullest equipment for the most advanced training and research in various branches of science, especially in its application to industry." Whatever misgivings might have been felt by those who were unaware of the new Rector's personality when the appointment was first announced, these rapidly vanished as a nearer acquaintance revealed the fact that the College had secured a most able administrator, and one endowed with a complete grasp of the things that matter and that make for progress. The College rapidly made headway under Sir Alfred's wise policy of encouraging initiative, for while keeping the reins of high policy well in hand, the new Rector succeeded in stimulating each and every one to contribute of his very best towards the common aim.

Anyone who is at all acquainted with the very special gifts required to run a group of federated institutions efficiently, while diminishing the friction almost inseparable from bringing together different bodies of men of distinct historical traditions, must realise that it needed a man of exceptional talent to act as the principal officer of such a federation. But it is the fact that Sir Alfred Keogh was able, and had gone a long way to achieve this object, when the outbreak of the war caused an interruption in his work which has endured for more than three years.

### UNIQUE QUALITIES IN EMINENT DEGREE.

Those who are most fully conversant with the medical aspect of the war are well aware of the invaluable services rendered by Sir Alfred Keogh in his capacity of acting Director-General of Army Medical Services, to which post he was appointed on the urgent representation of Lord Kitchener and others. It is through his untiring energy and his capacity of getting the right men to work on the main problems that our Armies in the Field have enjoyed such health as no army has ever known before. The secret of success lies in grasp, foresight, and in knowing what are the means available and how they shall be used. And these are qualities which are possessed by the Director-General in an eminent degree. He is now again leaving the Service, having brought into existence a machinery which works admirably and runs smoothly.

### DONE AND YET TO ACCOMPLISH.

But in exchanging his sphere of action and resuming his office as Rector of the Imperial College it is safe to say that Sir Alfred Keogh is obeying the call of duty. He has created a vast and thoroughly efficient Department at the War Office, which he will hand over to his successor there in perfect working order. His great administrative abilities are needed, and urgently needed, in the important task of organising science and industry—industry and science. For unless the threads of science are well woven into the warp of industry so that there is no longer separative, but real and integral communion, the future fate of this country would be sealed, whatever be the immediate outcome of the war. We in Britain have a great leeway to make up. Not only Germany, but America and other countries as well, were leaving us behind. It is because the country is waking up to the importance of science and of the furthering of scientific knowledge, both as directly and indirectly concerned with industry, that the largest College of its kind cannot, in the interests of the country, any longer be left without its principal officer. Sir Alfred Keogh is performing a patriotic part in deciding again to help to guide the industrial and scientific revolution

(none the less real, though perhaps it may escape the ken of the man in the street) which is actually going on in our midst. He thus acted three years ago, when the paramount necessities of the Empire called for an experienced and creative hand for the Department, on the efficient working of which so much of the safety of the Army depends. He will return to the Imperial College of Science and Technology with very real pleasure and fruitful expectations, for his heart is in the work. Its importance to the nation and Empire it would be difficult to exaggerate, and there is no one who could hope to accomplish, what is so urgently needed at the moment, with the grip and driving force of Sir Alfred Keogh. As the head of this great department of science he can render unique service by securing, with despatch and the maximum of efficiency results, without which, the future outlook of the country and Empire would be of the gravest.

